# Comparative Biological Half-Life of Penthiopyrad and Tebufenpyrad in Angelica Leaves and Establishment of Pre-Harvest Residue Limits (PHRLs)

**DOI:** 10.3390/foods13111742

**Published:** 2024-06-01

**Authors:** So-Hee Kim, Yoon-Hee Lee, Mun-Ju Jeong, Ye-Jin Lee, Hye-Ran Eun, Su-Min Kim, Jae-Woon Baek, Hyun Ho Noh, Yongho Shin, Hoon Choi

**Affiliations:** 1 Department of Applied Bioscience, Dong-A University, Busan 49315, Republic of Korea; 2271775@donga.ac.kr (S.-H.K.);; 2 Residual Agrochemical Assessment Division, National Institute of Agricultural Sciences, Wanju 55365, Republic of Korea; 3 Department of Life & Environmental Sciences, Wonkwang University, Iksan 54538, Republic of Korea

**Keywords:** LC-MS/MS, PHRL, angelica leaves, penthiopyrad, tebufenpyrad, QuEChERS

## Abstract

To prevent pesticides from exceeding maximum residue limits (MRLs) in crops during export and shipment, it is necessary to manage residue levels during the pre-harvest stages. Therefore, the Republic of Korea establishes pre-harvest residue limits (PHRLs) per crop and pesticide. This study was conducted to set PHRLs for penthiopyrad and tebufenpyrad in angelica leaves, where the exceedance rates of MRLs are expected to be high. The LOQ of the analytical method used was 0.01 mg/kg and it demonstrated good linearity, with a correlation coefficient of 0.999 or higher within the quantitation range of 0.005 to 0.5 mg/kg. The recovery and storage stability accuracy values were in the range of 94.5–111.1%, within the acceptable range (70–120%, RSD ≤ 20%). The matrix effect for both pesticides was in the medium-to-strong range, and it did not significantly impact the quantitative results as a matrix-matched calibration method was employed. Using the validated method, residue concentrations of penthiopyrad 20 (%) EC and tebufenpyrad 10 (%) EC were analyzed. Both pesticides exhibited a decreasing residue trend over time. In Fields 1–3 and their integrated results, the biological half-life was within 2.6–4.0 days for penthiopyrad and 3.0–4.2 days for tebufenpyrad. The minimum value of the regression coefficient in the dissipation curve regression equation was selected as the dissipation constant. The selected dissipation constants for penthiopyrad in Fields 1–3 and their integration were 0.1221, 0.2081, 0.2162, and 0.1960. For tebufenpyrad, the dissipation constants were 0.1451, 0.0960, 0.1725, and 0.1600, respectively. The dissipation constant was used to calculate PHRL per field. Following the principles of the PHRL proposal process, residue levels (%) on PHI dates relative to MRLs were calculated, and fields for proposing PHRLs were selected. For penthiopyrad, since the residue level (%) was less than 20%, the PHRL for Field 3 with the largest dissipation constant was proposed. For tebufenpyrad, as the residue level (%) exceeded 80%, the PHRL proposal could not established. It is deemed necessary to reassess the MRL and ‘guidelines for safe use’ for tebufenpyrad in angelica leaves.

## 1. Introduction

In the ‘2022 Consumer Behavior Survey for Foods’ by the Korea Rural Economic Institute, over half of respondents were worried about pesticide residues in crops [1]. This indicates a high level of apprehension among consumers regarding pesticide residues. There is a recognized need for systematic safety management concerning pesticide residues in crops.

The Republic of Korea’s Ministry of Food and Drug Safety (MFDS) sets Pre-Harvest Residue Limits (PHRLs) to proactively address potential exceedances of Maximum Residue Limits (MRLs) in crops during pre-harvest steps [2]. The pre-harvest steps encompass the overall period from crop growth to development. PHRL refers to the maximum allowable concentration of pesticides in the pre-harvest steps, by date (up to 10 days before shipment). Exceeding the PHRL during pre-harvest steps results in actions such as disposal, shipment delay, and purpose conversion [3]. The selection of crops and pesticides for PHRL setting reflects the standards as determined by the National Agricultural Products Quality Management Service. Pesticides and crops with a high frequency of unsuitability in the past three years are also included.

To ensure the supply of safe crops, it is necessary to identify crops and pesticides that may exceed MRLs during the pre-harvest step and take preemptive measures. In the Republic of Korea, the number of registered MRLs stands at 12,422 (As of January 2024), whereas the registered PHRLs are relatively fewer, totaling 1241 [4]. Additional research on PHRLs is needed for numerous crops, including minor crops.

Angelica is primarily consumed in Korea, China, and Japan, and is classified based on its origin as Korean angelica (*Angelica gigas* Nakai), Japanese angelica (*Angelica acutiloba* Kitagawa), and Chinese angelica (*Angelica sinensis (Oliv.)* Diels) [5]. The pharmacological effects of angelica varies depending on its origin [6]. The roots of angelica are mainly used for medicinal purposes, while the leaves are utilized as leafy vegetables [7]. Among these angelica varieties, the one used for leafy vegetables is Japanese angelica. The leaves of Japanese angelica contain components such as falcarindiol, bergaptol, and bergaptol, which exhibit anti-inflammatory activity [8]. In the Republic of Korea, angelica has traditionally been used as a medicinal herb and has been documented in many medical texts [9,10]. Angelica is widely exported not only domestically but also internationally. Therefore, angelica is considered a culturally, historically, and economically important crop in the Republic of Korea [11,12,13]. The consumption of angelica leaves, in addition to its roots, has recently been increasing [14]. Angelica leaves are used as a vegetable due to their unique fragrance and flavor [15].

**Figure 1:**
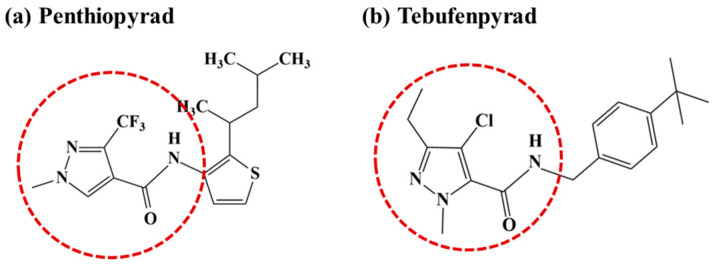
Structural formulas of pyrazolecarboxamide-based pesticides. (**a**) Penthiopyrad and (**b**) tebufenpyrad. Dotted circles refer to the structure of the pyrazolecarboxamide series.

Pyrazolecarboxamides, acknowledged for their high efficiency and low toxicity, are systemic pesticides. They also demonstrate excellent compatibility with humans and beneficial organisms, making them environmentally friendly pesticides [16]. Penthiopyrad and tebufenpyrad, two representative pyrazolecarboxamide-based pesticides, were developed in Japan in 2009 and 1992, respectively [17,18,19,20]. Penthiopyrad, a succinate dehydrogenase inhibitor (SDHI) fungicide [21,22], is used to control gray mold, powdery mildew, and other diseases [23]. Tebufenpyrad serves as a mitochondrial electron transport inhibitor (METI) acaricide and insecticide [24]. It is primarily used for the control of two-spotted spider mites and other pests [25]. The structures of these pesticides are depicted in Figure 1. Pyrazolecarboxamide-based insecticides contain the pyrazol-5-carboxamide moiety, while fungicides contain the thepyrazol-4-carboxamide motif [26].

Penthiopyrad and tebufenpyrad in angelica leaves, with established MRLs and ‘guidelines for the safe use’, are utilized in various pesticide products. PHRLs for both pesticides have not been established in angelica leaves. PHRLs are established based on conditions when the residual concentration of pesticides is expected to be the highest, for consumer safety, selecting the pesticide product with the highest active ingredient level and anticipated high residue and ensuring it meets the MRLs and ‘guidelines for safe use’. For this study, the experimental fields selected were greenhouses with a high likelihood of pesticide residue in their crops. The crop cultivation period was chosen to conduct trials during a time of elevated rates of unsuitable pesticide levels. 

To establish the PHRLs for penthiopyrad 20 (%) emulsifiable concentrate (EC) and tebufenpyrad 10 (%) EC, residue studies were conducted in angelica leaf fields. The analysis of active ingredients was performed using validated methods, ensuring accuracy and precision. This study can serve as foundational data for setting PHRLs for penthiopyrad and tebufenpyrad in angelica leaves.

## 2. Materials and Methods

### 2.1. Chemicals and Reagents

Penthiopyrad (purity 99.4%) and tebufenpyrad (98.0%) reference standards and 10 M of ammonium formate were purchased from Sigma-Aldrich (St. Louis, MO, USA). HPLC grade acetonitrile (MeCN) was obtained from Duksan Pure Chemical (Seoul, Republic of Korea). Formic acid (99.0%) and LC-MS grade methanol were sourced from Thermo Fisher Scientific (Waltham, MA, USA), while LC-MS grade water was acquired from Merck (Darmstadt, Germany). A QuEChERS extraction pouch (EN 15662) containing 4 g of magnesium sulfate (MgSO_4_), 1 g of sodium chloride (NaCl), 1 g of sodium citrate (Na_3_Citr·2H_2_O), and 0.5 g of sodium hydrogen citrate sesquihydrate (Na_2_HCitr·1.5H_2_O) and dispersive SPE (d-SPE) containing PSA 25 mg and MgSO_4_ 150 mg were purchased from Agilent Technologies (Santa Clara, CA, USA). Commercial pesticide products were Cleancap, penthiopyrad 20 EC (produced by Kyung Nong Co., Ltd., Seoul, Republic of Korea) and Piranika, tebufenpyrad 10 EC (manufactured by Syngenta Korea Co., Ltd., Seoul, Republic of Korea). For the field trial, both products were diluted to 2000 times (e.g., 10 mL product/20 L total solution). The temperature and humidity in fields were measured using a Thermo Recorder TR72wb thermohygrometer from T&D Corporation (Matsumoto, Japan).

### 2.2. Field Trial

**Figure 2:**
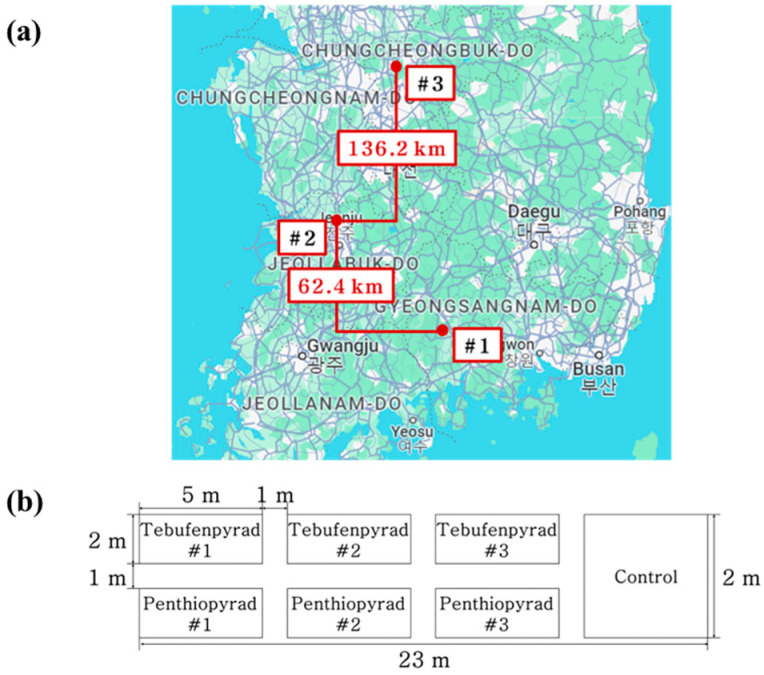
Experimental field planning. (**a**) Field location selection and (**b**) layout of the fields. In (**a**), #1 is Field 1 (Sancheong), #2 is Field 2 (Jeonju), and #3 is Field (Jecheon).

Field trials were conducted in three different locations, including Sancheong-gun, Gyeongsangnam-do (Field 1); Jeonju-si, Jeollabuk-do (Field 2); and Jecheon-si, Chungcheongbuk-do (Field 3), Korea. To evaluate residue characteristics based on climate variations, each field was selected in regions with a latitude difference of over 20 km (Figure 2a). The treatment groups were designed with three replicates, while the control group had one replicate for each target pesticide. The layout of the experimental fields is depicted in Figure 2b. Buffer zones of at least 1 m were arranged between the treated replicate areas. The designated area for each treatment group was a minimum of 10 m^2^. The same variety of angelica leaves (*Angelica acutiloba* Kitagawa) was used in all fields and the planting density was set at 30 × 30 cm.

The field trial was carried out following the ‘guidelines for safe use’ established by the Rural Development Administration of Korea. Both pesticides have a pre-harvest interval (PHI) of 7 days, with penthiopyrad allowing up to 3 applications and tebufenpyrad limited to once during this period before harvest. Penthiopyrad 20 EC and tebufenpyrad 10 EC were diluted 2000-fold and applied as a foliage treatment at a dose of 120 L/10 a. Pesticides were applied using a backpack sprayer, EL969-2 (Field 1) from Perfect-EL (Siheung, Korea) and MSB1500Li (Fields 2 and 3) from Maruyama (Tokyo, Japan).

Angelica leaves can be harvested one month after planting. Field 1 and 3 were planted in March 2023, and Field 2 was planted in February 2023. Samples were collected in quantities of at least 1 kg at 0 (within 2 h after treatment), 1, 2, 3, 5, 7, 10, and 14 days following pesticide applications. The samples were stored in a deep freezer (−80 °C) until they were prepared. 

### 2.3. Standard Solutions and Calibration Curves

Stock solutions (1000 mg/L) were prepared by dissolving each standard in MeCN. These solutions were then diluted with MeCN to create a working solution with a concentration of 20 mg/L, which was subsequently serially diluted to concentrations of 0.5, 0.25, 0.1, 0.05, 0.025, 0.01, and 0.005 mg/L. For the calibration curve plotting, individual working solutions (300 μL) were mixed with 300 μL of extracts of pesticide-free samples to produce matrix-matched standard solutions with concentrations of 0.25, 0.125, 0.05, 0.025, 0.0125, 0.005, and 0.0025 mg/L. Two microliters of penthiopyrad and five microliters of tebufenpyrad matrix-matched standards were injected into liquid chromatography-tandem mass spectrometry (LC-MS/MS) and the correlations were verified between peak areas and concentrations in the chromatograms.

### 2.4. Analytical Conditions for LC–MS/MS

The analysis was conducted using a Shimadzu (Kyoto, Japan) LCMS-8040 triple quadrupole mass spectrometer, connected to a Shimadzu Nexera liquid chromatograph. The chromatograph setup included a degasser (DGU-20A5), pump (LC-30AD), auto sampler (SIL-30AC), communications bus module (CBM-20A), and column oven (CTO-20A).

The chromatographic separation utilized a Kinetex PS C18 column (2.6 μm, 3 × 100 mm) from Phenomenex (Torrance, CA, USA). Mobile phase A was composed of 5 mM ammonium formate and 0.1% formic acid in water, while mobile phase B contained 5 mM ammonium formate and 0.1% formic acid in methanol. For penthiopyrad, the mobile phase B gradient initiated at 30% (0.0–0.2 min) and then escalated to 90% (0.2–0.5 min). It was further raised to 98% (0.5–6 min) and maintained (6–9 min). Subsequently, it was lowered to 30% (9–9.1 min) and allowed to equilibrate (9.1–13 min) at the initial percentage ratio. In the case of tebufenpyrad, on the other hand, increased to 80% at 0.2–0.5 min, while the other conditions remained the same. The column oven temperature was set at 40 °C and the flow rate was adjusted to 0.2 mL/min. Penthiopyrad and tebufenpyrad were injected at volumes of 2 μL and 5 μL, respectively.

In the mass spectrometry conditions, the analysis of the target pesticides was conducted using positive electrospray ionization (ESI+) and multiple reaction monitoring (MRM) modes. For collision-induced dissociation (CID), argon gas with a purity of 99.999% was utilized. The desolvation line (DL) and heat block temperatures were set at 250 °C and 400 °C, respectively. Additionally, the flow rates for the drying gas and nebulizing gas were maintained at 15 and 3 L/min, respectively. The processing of data was carried out using Shimadzu Lab-Solutions software, version 5.60 SP2.

To establish the MRM conditions, a full scan mode was employed, analyzing a mass-to-charge ratio (*m*/*z*) range from 150 to 800 at a rate of 410 u/s and selecting each precursor ion of the pesticide. Confirmation of precursor ion fragmentation was achieved by conducting a product ion scan with varying collision energies (CE). The quantifier and qualifier ions of MRM transitions and their respective CEs were optimized based on sensitivity and selectivity.

### 2.5. The Preparation Method

A homogenized sample, weighing 10 g, was mixed with 10 mL of MeCN. The sample was subjected to a 2 min extraction at 1300 rpm using a Genogrinder (1600 Mini-G, SPEX SamplePrep, Metuchen, NJ, USA) and 4 g of MgSO_4_, 1 g of NaCl, 1 g of Na_3_Citr·2H_2_O, and 0.5 g of Na_2_HCitr·1.5H_2_O were added. It was shaken for 1 min at 1300 rpm and then centrifuged for 5 min at 3500 rpm using a centrifuge (M15R, Hanil Scientific, Gimpo, Republic of Korea). One milliliter of supernatant was combined with 25 mg PSA and 150 mg MgSO_4_ in a dSPE tube, then vortexed for 1 min and centrifuged at 13,000 rpm for 5 min. Then, 300 μL supernatant was mixed with 300 μL of MeCN and injected into a LC-MS/MS system.

### 2.6. Method Validation

Several concentrations of standard solutions were analyzed using LC-MS/MS, and the lowest concentration was selected as the limit of quantitation (LOQ) from among those satisfying the signal-to-noise ratio (S/N ≥ 10) and accuracy and precision (recovery rate 70–120%, RSD ≤ 20%) requirements. 

The recovery study was conducted at 0.01 mg/kg, 0.1 mg/kg, MRL levels (penthiopyrad, 15 mg/kg; tebufenpyrad, 1 mg/kg), and the highest residue concentration level. Control samples weighing 10 g were spiked with a stock solution (1000 mg/L) or standard solutions to generate the recovery samples. The storage stability study utilized control samples collected in the PHI (7 days after the final application) from Field 1 (Sancheong) for both pesticides. The control samples of 10 g were spiked with a 100 mg/L standard solution to achieve a concentration of 1 mg/kg and then stored until analysis. The samples were prepared, followed by matrix-matching and analysis using LC-MS/MS.

### 2.7. Dilution Effect of Penthiopyrad and Tebufenpyrad in Angelica Leaves

The residual concentrations of penthiopyrad and tebufenpyrad, accounting for daily weight changes in the angelica leaves, were calculated for dilution effects (A) using Equation (1). Residue concentrations, excluding dilution effects (B), were calculated with Equation (2). The dilution effect due to changes in the weight of the crops was referenced from the studies of [27,28,29].

(1) $$(A)=\frac{Residue\;concentrations\;on\;day\;0\;\times \;The\;average\;weight\;of\;crops\;on\;day\;0\;(n=3)}{The\;average\;weight\;of\;crops\;by\;harvest\;date\;(n=3)}$$

(2) $$\left(B)=Pesticide\;residue\;concentrations\;on\;day\;0\;-\;\{\left(A\right)\;-\;Pesticide\;residue\;concentrations\;by\;harvest\;date\right\}$$

### 2.8. Calculation of Biological Half-Life and PHRL

Based on the residue concentrations by harvest date of pesticides in angelica leaves, dissipation curves were plotted. Using the dissipation regression equation derived from the curve, regression coefficients and biological half-lives were calculated. The dissipation regression equation was utilized as follows:

(3) $$C_{t}=C_{0}e^{-kt}$$

where *C*_0_ is the concentration at 0 day, *k* is the regression coefficient, and *t* is days after pesticide spraying.

The biological half-life was calculated by incorporating the dissipation regression equation into Equation (4).

(4) $$t=\frac{ln2}{k}=\frac{0.693}{k}$$

The significance of the regression equation and regression coefficient was verified through F-test and *T*-test. The regression equation and coefficients were verified for significance at a 95% confidence level using F-test and *T*-test. The 95% confidence interval (CI) for the regression coefficients was calculated, and its minimum value was selected as the dissipation rate constant. The PHRLs were calculated by date, substituting values into Equation (5).

(5) $$PHRL\;by\;date=MRL\;\times \;e^{Dissipation\;rate\;constant\times Pre-harvest\;date}$$

## 3. Results and Discussion

### 3.1. The Established MRM Conditions and Method Validation

The monoisotopic masses for penthiopyrad and tebufenpyrad are 359.1 and 333.2 g/mol, respectively. In full scan mode, their m/z values were found to be 360.1 and 334.2. It was determined that both pesticides were ionized as [M + H]^+^. This ionization pattern could also be observed in a mass spectrometry study based on liquid chromatography [30,31]. Product ion scanning was performed by applying CEs from −10 V to −100 V at −10 V intervals. The most sensitive and selective ion in the product ion scan spectrum was chosen as the quantifier, with the second ion selected as the qualifier. Based on the CE with the highest sensitivity for quantitation and qualification ions (range of −10 to −100 V), a more detailed CE was applied for MRM analysis. From the result, the CE at the highest point on the graph was selected as the optimal CE for establishing the MRM conditions. MRM conditions and retention times for the pesticides are provided in Table 1.

The method’s reliability was verified through LOQ, calibration linearity, recovery, and storage stability. Both penthiopyrad and tebufenpyrad exhibited an LOQ of 0.005 mg/L, which is the lowest concentration of standard solution where S/N exceeded 10 (*n* = 4). The calibration curve ranged from 0.0025–0.25 mg/L, which corresponds to 0.005–0.5 mg/kg in angelica leaves. No weighting factor was utilized. The correlation coefficients (*r*^2^) of 0.9999 were observed for both pesticides, indicating a satisfactory level of linearity.

**Figure 3:**
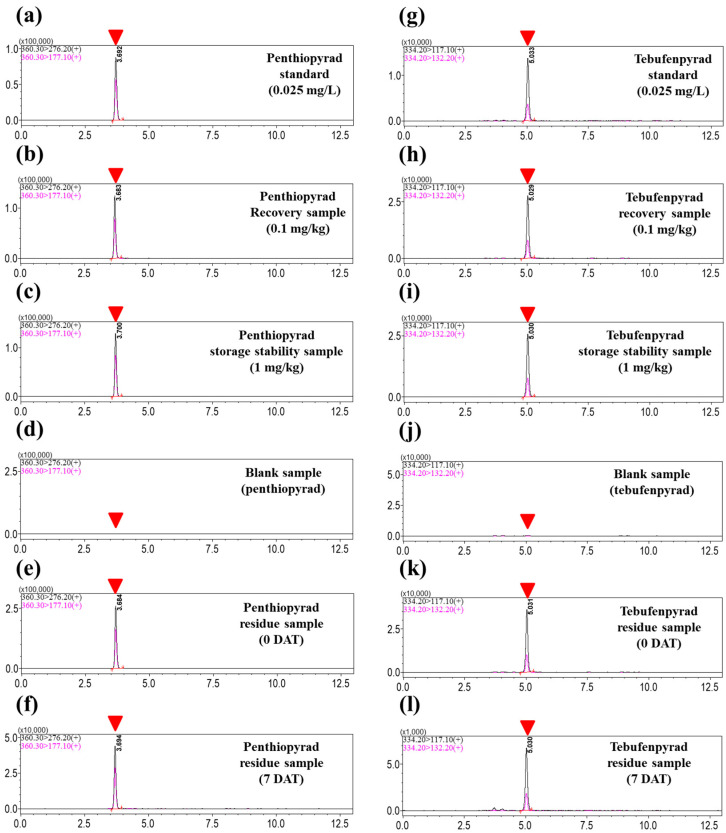
Chromatograms for pesticides. (**a**) Penthiopyrad matrix-matched standard at 0.025; (**b**) penthiopyrad recovery sample at 0.1 mg/kg; (**c**) penthiopyrad storage stability sample at 1 mg/kg; (**d**) blank sample without penthiopyrad; penthiopyrad residue sample at (**e**) 0 DAT and (**f**) 7 DAT; (**g**) tebufenpyrad matrix-matched standard at 0.025; (**h**) tebufenpyrad recovery sample at 0.1 mg/kg; (**i**) tebufenpyrad storage stability sample at 1 mg/kg; (**j**) blank sample without tebufenpyrad; tebufenpyrad residue sample at (**k**) 0 DAT and (**l**) 7 DAT. The red arrowhead indicates the peak detected at the retention time for each pesticide. The blank sample shows the retention time since no peaks were detected.

The recovery study was conducted at 0.01 and 0.1 mg/kg, as well as at the concentrations of the MRL. Following the SANTE guidelines (70–120% with RSD ≤ 20%) [32], the results showed acceptable average values for penthiopyrad; 104.1, 94.5, and 99.6% (with RSD ≤ 2.0%), and for tebufenpyrad; 107.1, 108.1, and 104.1% (with RSD ≤ 2.6%). The tebufenpyrad recovery at 20 mg/kg, which exceeded the highest calibration point, was additionally confirmed due to the residue study. The dilution method for tebufenpyrad demonstrated accuracy by achieving a recovery rate of 111.4% (RSD 2.0%). The storage stability study was performed to assess potential changes, such as degradation or transformation of the pesticides, that could arise during sample storage (Table 2). Storage stability was determined at a level of 1 mg/kg. The target pesticides exhibited stability in angelica leaves when stored at a frozen temperature of −20 °C. The recovery rates were 97.0% (RSD 9.1%) for penthiopyrad and 105.5% (RSD 3.2%) for tebufenpyrad. Figure 3 shows chromatograms for penthiopyrad and tebufenpyrad.

### 3.2. Temperature and Humidity in Field

During the experimental period, the average temperatures for Fields 1 to 3 were 20.7 ± 3.8 °C, 19.1 ± 4.2 °C, and 17.5 ± 4.2 °C, respectively. The average humidities were 66.2 ± 12.9%, 65.1 ± 11.3%, and 72.6 ± 13.0% for each field.

### 3.3. Variations in Sample Weight over Date

For each day after treatment (DAT), 30 angelica leaves were bundled together, and the weight of three bundles was averaged. In the case of the penthiopyrad treatment groups, the average weight of angelica leaves exhibited a range of 71.2 ± 0.6 g to 79.2 ± 0.7 g in Field 1, 72.1 ± 0.7 g to 79.2 ± 1.0 g in Field 2, and 70.2 ± 1.8 g to 75.8 ± 0.6 g in Field 3. For the tebufenpyrad treatment groups, the leaves’ weight range was 71.0 ± 1.5 g to 78.9 ± 0.2 g in Field 1, 71.2 ± 0.8 g to 80.7 ± 1.3 g in Field 2, and 69.8 ± 1.3 g to 73.7 ± 0.4 g in Field 3. All samples were selected and collected in a distributable growth state. Uniform-sized angelica leaves were harvested by date to minimize pesticide dilution effects due to growth in diameter.

### 3.4. Pesticide Residue Characteristics at the Pre-Harvest Phase of Angelica Leaves

**Figure 4:**
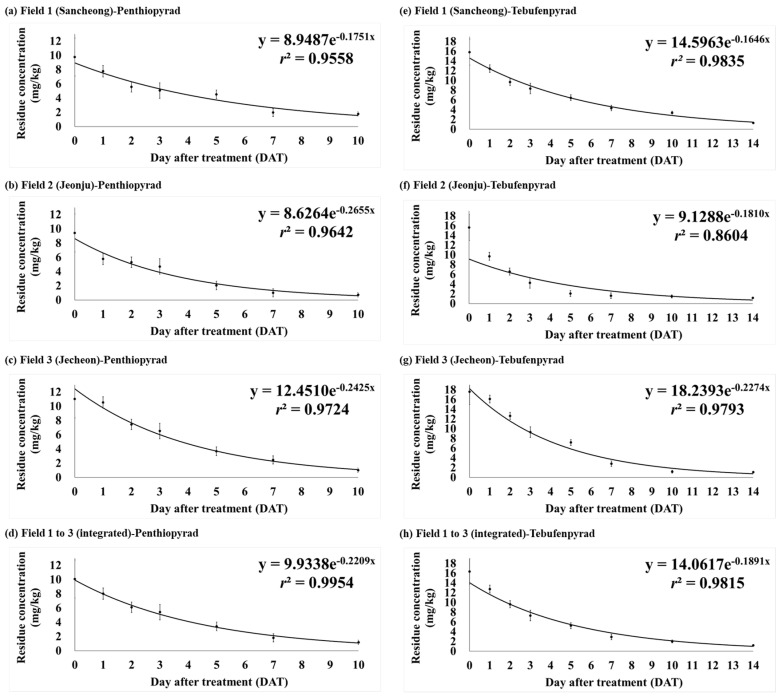
The dissipation curves of pesticide in each field and their integrated results. Sub-figures (**a**–**d**) are for penthiopyrad and (**e**–**h**) are for tebufenpyrad.

In order to evaluate the residue characteristics of penthiopyrad and tebufenpyrad in angelica leaves, the analysis was carried out after pesticide application in accordance with the ‘guidelines for safe use’. The changes in residue amount by date and the average residues at the three fields are presented in Table 3. The dissipation curve is plotted in Figure 4. In Fields 1 to 3, the initial concentrations after the final pesticide spraying were 9.77, 9.43, and 11.02 mg/kg for penthiopyrad and 15.82, 15.61, and 17.60 mg/kg for tebufenpyrad, respectively. 

**Figure 5:**
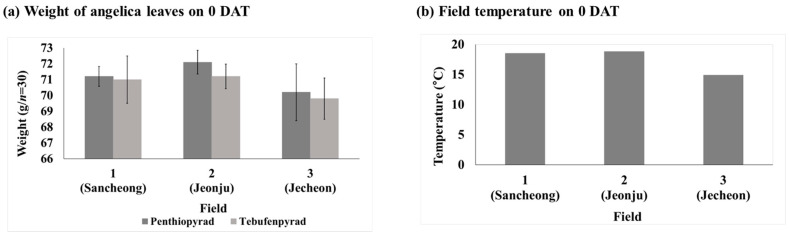
Weight and temperature of each field at 0 DAT. (**a**) Weight of angelica leaves and (**b**) field temperature.

Both pesticides exhibited similar initial concentrations in Fields 1 and 2, with slightly elevated concentrations observed in Field 3. This tendency is attributed to stem from the smaller growth status of the leaves on day 0 in Field 3 compared to the other fields, coupled with the lower temperature (Figure 5). The factors affecting the longevity of pesticides in crops are varied and encompass the following: geographic regions and climatic conditions of the cultivation area, purpose, composition, and method of pesticide application, etc. [33]. In the research conducted by Saini et al., residue studies were conducted for spirodiclofen and tebufenpyrad on Aster scaber in two different fields. In Field 1, the residue amount of spirodiclofen was 14.2–3.0 mg/kg, while in Field 2, it was 8.9–1.5 mg/kg. Tebufenpyrad showed residue amounts of 4.9–0.8 mg/kg in Field 1 and 2.0–0.4 mg/kg in Field 2. For both pesticides, the initial residue levels (0 DAT) were higher in Field 1 than Field 2. The average weight of the crops ranged from 131.0 ± 9.2 g in Field 1 to 137.3 ± 6.1 g in Field 2 at 0 DAT. The temperature was lower in Field 1 than in Field 2. Therefore, it is presumed that in the preceding study, as well as for the relatively higher initial residue levels in Field 1, the results were influenced by crop growth and temperature.

For penthiopyrad, the residue concentration on the 7th day of the PHI remained below the MRL of 15 mg/kg in all three fields. However, for tebufenpyrad, in Fields 1–3, the average residue concentration was 4.36, 1.61, and 2.81 mg/kg, sequentially, all of which exceeded the MRL of 1 mg/kg across the three fields. In previous studies, cases were reported where tebufenpyrad exceeded the MRL in apples [34]. Therefore, the reassessment of pesticide MRLs or ‘guidelines for safe use’ may be necessary for tebufenpyrad. 

**Figure 6:**
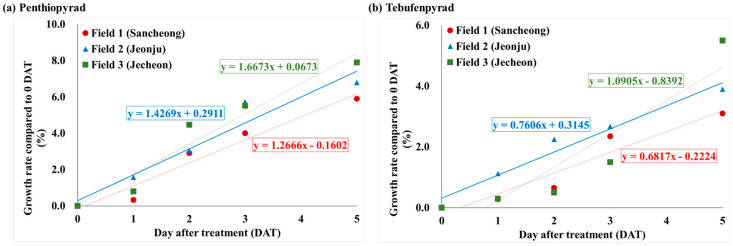
After the final pesticide application, the growth rate of angelica leaves in each field during the initial period (0−5 DAT) for (**a**) penthiopyrad and (**b**) tebufenpyrad.

Variations in biological half-life can arise due to diverse factors, including the formulation type of the pesticide, crop variety, field temperature or humidity levels, and metabolic processes [35]. Additional research is needed in controlled environments to identify precise determinations [36]. The half-life of penthiopyrad, calculated using the regression coefficient, for daily pesticide residue in angelica leaves was 4.0 days in Field 1, 2.6 days in Field 2, 2.9 days in Field 3, and 3.1 days in their integrated results. For tebufenpyrad, the biological half-life was 4.2 days, 3.8 days, and 3.0 days in Field 1–3, respectively, with an integrated time of 3.7 days. Both penthiopyrad and tebufenpyrad exhibited a slightly longer half-life tendency in Field 1 compared to Fields 2 and 3. The initial crop growth rate after the final application of the pesticide (0–5 DAT) was calculated and depicted graphically (Figure 6). The slope of the growth rate line in Fields 1–3 for penthiopyrad was 1.2666, 1.4269, and 1.6673, respectively. For tebufenpyrad, the slope was 0.6817 in Field 1, 0.7606 in Field 2, and 1.0905 in Field 3. Field 1 showed the gentlest slope for both pesticides. This implies that in Field 2 and 3, angelica leaves grew faster than Field 1 between days 0 and 5. It was determined that the longest half-life, in Field 1, was influenced by the crop’s growth rate [37]. In Field 1, the growth rate during the initial period after the final pesticide application was slower compared to the other fields, resulting in relatively smaller leaf sizes. Therefore, it was determined that the dilution effect from crop growth was less pronounced. In previous studies on the half-life of penthiopyrad in crops, it has been reported as 10.2 days in peucedanum japonicum [38], 2.5–2.6 days in cucumber [39], and 2.7–3.0 days in oriental melon [40]. For tebufenpyrad, the half-life has been reported as 3.8–4.2 days in aster scaber and 3.5–3.7 days in perilla leaves [41]. In angelica leaves, penthiopyrad demonstrated a half-life similar to that seen in cucumber and oriental melon, while tebufenpyrad exhibited a trend similar to the one seen in aster scaber and perilla leaves. To compare the half-lives of the same pesticide in different crops, it is judged that the research should be conducted under fixed conditions.

### 3.5. Verifying Pesticide Dilution Effects from Increased Weight of Angelica Leaves

**Figure 7:**
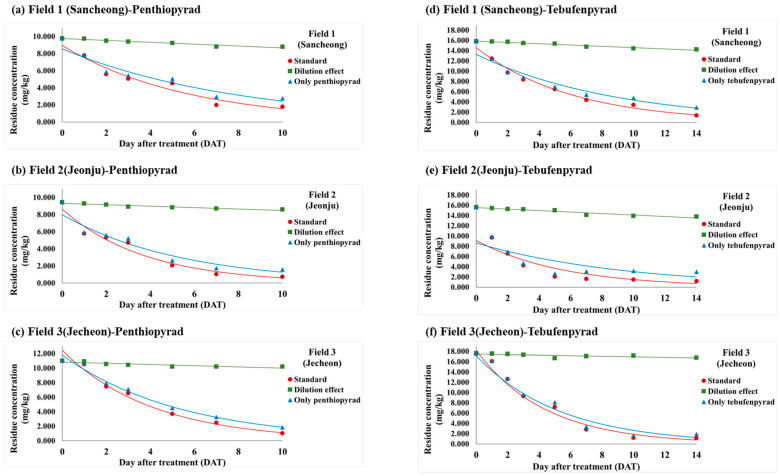
Dilution effects due to diameter growth and penthiopyrad residual dissipation curves reflecting the dilution effect. Graphs (**a**,**d**) represent data from Field 1; (**b**,**e**) are from Field 2, and (**c**,**f**) are from Field 3. (**a**–**c**) are the graphs for penthiopyrad and (**d**–**f**) are the graphs for tebufenpyrad. ‘Standard’ refers to the original residue concentration without accounting for the dilution effect. ‘Only pesticide’ refers to the residue concentration accounting for the dilution effect.

The residue amount and pattern of pesticides in crops are influenced by the formulation, bioavailability, and efficacy of the pesticide [42]. Among these factors, the dilution effect of pesticides based on the growth in diameter of the crops was investigated. The dilution effect on the weight changes of angelica leaves considered, the average residue amount of penthiopyrad was 2.740–9.773 mg/kg in Field 1, 1.585–9.430 mg/kg in Field 2, and 1.797–11.016 mg/kg in Field 3. For tebufenpyrad, the average residue amount was 2.788–12.478 mg/kg, 2.891–9.861 mg/kg, and 2.084–16.208 mg/kg in Fields 1–3, sequentially. The residue levels of the existing pesticide (including dilution effects), only pesticide (excluding dilution effects), and dilution effect are depicted in Figure 7. 

When reviewing previous studies on the dilution effect based on crop growth, a study on bifenthrin application in grapes showed that the weight increased by 29.6% up to 15 days after spraying compared to day 0 [27]. The difference in residual pattern between the pesticide dissipation curve, inclusive and exclusive of the dilution effect, was low. In the study of bistrifluron and cyenopyrafen in peaches by Hwang [29], the weight increased by 21.6–30.6% from days 0 to 14. Additionally, in research on the dilution effect of amisulbrom in winter-grown cabbage by Ahn [28], there was a weight increase of 50.6–53.4% between days 0 and 10. In these studies, similar dissipation patterns were observed, both when accounting for dilution effects and when not. It was concluded that the dilution effect due to growth in crop diameter was minimal. In Lee’s study on the dilution effect of the fungicide boscalid in cucumbers, known for their rapid growth, the crops had increased in weight by approximately 16 times on the 10th day compared to the initial day [43]. It was concluded that the pesticide residue dissipation curve, excluding the dilution effect, showed almost no decrease, indicating a dilution effect due to the increase in growth in diameter. Cucurbitaceous fruit vegetables such as cucumber and zucchini are characterized by a rapid increase in growth rate. In a study on pesticide residues in cucurbitaceous fruit vegetables [44], vigorous crop growth, including for Korean zucchini and zucchini, led to a dilution effect for bifenthrin and bitertanol.

The test crop, angelica leaves, in this study did not exhibit rapid growth rates akin to cucurbitaceous crops, showing a weight difference within 12.4% across all fields. Compared to grapes, peaches, and winter-grown cabbage, this is a low growth rate, and the difference between the daily residue curves and those excluding the dilution effect is low. Therefore, it is concluded that the dilution effect due to the growth in diameter of the crop is low in angelica leaves.

### 3.6. PHRL Calculation and Proposal

**Figure 8:**
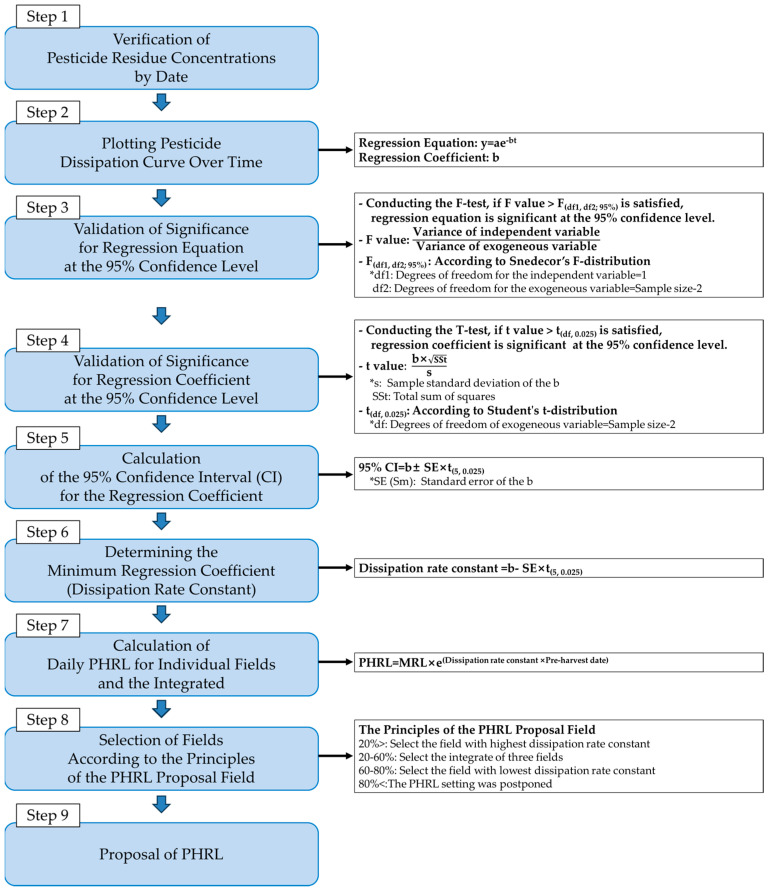
Process of proposing a PHRL.

The proposal of PHRLs was conducted following the process in the diagram in Figure 8 [45].

F-testing was conducted to assess the significance of the regression equation for the dissipation curve. The regression equation was considered significant at a 95% confidence level if, after the analysis of variance, the F-value > F_(df1, df2; 95%)_. ‘df1’ denotes the degrees of freedom for the independent variable and ‘df2’ represents the degrees of freedom for the exogenous variable. If t > t_(df, 0.025)_ in the *T*-test, the regression coefficient was considered to have a confidence level of 95% or higher. The dissipation constant was determined as the minimum value of the regression coefficient within the 95% confidence interval.

In F-tests, the ‘df1’ was 1 for both pesticides. The ‘df2’ for penthiopyrad was 5, and for tebufenpyrad, it was 6. According to the F-distribution, F_(1,5;95%)_ was 6.6079 and F_(1,6;95%)_ was 2.4469. In Fields 1–3 and their integrated results, for penthiopyrad and tebufenpyrad, the F-values ranged from 71.2315 to 559.7037 and 27.1257 to 424.1372, respectively. In all fields and averages of both pesticides, F-value > F_(df1,df2;95%)_ was satisfied, and the change in residual amounts over time was judged to have a significant regression relationship at the 95% confidence level. For the *T*-testing, the ‘df’ was 5 for penthiopyrad and 6 for tebufenpyrad. For the T-distribution, t_(5, 0.025)_ equaled 2.5706, while t_(6, 0.025)_ was 2.4469. The penthiopyrad T-values ranged from 8.4989 to 23.6581 in Fields 1–3 and their averages. For tebufenpyrad, the T-value ranged from 5.2082 to 20.5946. In both pesticides, T-values were greater than t_(df,0.025)_, leading to the conclusion that the regression coefficients exhibited a confidence level of 95% or higher. Considering the dissipation rate constants and t_(df,0.025)_, confidence ranges for the regression coefficients were calculated. For penthiopyrad, the confidence ranges for the regression coefficients in Fields 1–3 were 0.1221–0.2280, 0.2081–0.3230, and 0.2162–0.2689, respectively. For tebufenpyrad, the range in Field 1 was 0.1451–0.1842, in Field 2 was 0.0960–0.2660, and in Field 3 was 0.1725–0.2823. The 95% CIs for the average residue levels in the three fields were 0.1960–0.2457 for penthiopyrad and 0.1600–0.2182 for tebufenpyrad (Table 4).

**Figure 9:**
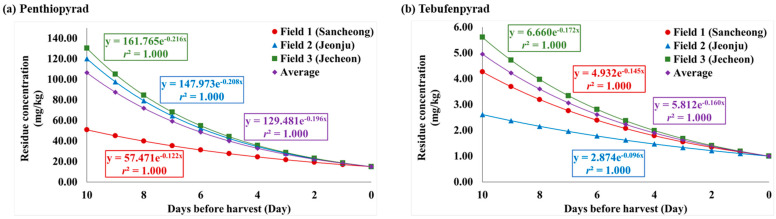
PHRL curve of each field and their integrated average. (**a**) Penthiopyrad and (**b**) tebufenpyrad.

The Pre-Harvest Residue Limit (PHRL) is a standard set from 10 days before harvest to prevent the distribution of agricultural products exceeding their MRLs at the time of harvest [46]. The daily PHRLs for penthiopyrad and tebufenpyrad in angelica leaves, calculated with the minimum value of regression coefficient in this study (Table 5), and the PHRL curve is presented in Figure 9. The calculation of the residue level (%) was based on Equation (6), using the average residue amount from the three fields at the PHI date and the MRL for each pesticide.

(6) $$Residue\;ratio=\frac{MRL}{Residue\;concentration\;by\;PHI\;date}\times 100$$

The choice of fields proposed for PHRLs accorded to the following principles: The residue level (%) is <20%; the field with highest minimum value of regression coefficient, 20–60%; the integrated results of the three fields, 60–80%; and the field with lowest minimum value of regression coefficient was selected. If the residue level (%) was >80%, the standard setting was postponed, and it was determined that a reevaluation of the MRL or ‘guidelines for safe use’ was necessary [47]. 

For penthiopyrad, the 1.82 mg/kg residue concentration was detected on the 7th day (PHI), based on the average residue amount from all three fields. The MRL for penthiopyrad is 15 mg/kg, and the residue level (%) is calculated to be 12.1% according to Equation (6). In accordance with the principles of PHRL proposal, if the residue level (%) is <20%, the PHRL of the field with highest minimum value of regression coefficient is applied. Therefore, the PHRL proposal is based on Field 3, which has the highest minimum value of regression coefficients. If the residue level is below 130.31 mg/kg 10 days before harvest or 44.21 mg/kg 5 days before, it is predicted that the penthiopyrad residue concentration will remain below the MRL when harvesting angelica leaves. Tebufenpyrad was detected at the average residue level of 2.93 mg/kg in the three fields on the date of the PHI. The MRL for tebufenpyrad is 1 mg/kg, and the residue level on the date of PHI exceeded the MRL by 293.0%, indicating a very high residue level. The residue level is >80%, and since all three fields exceeded the MRL levels at both the PHI and 14 DAT, the establishment of standards is pending in accordance with the principles of PHRL proposal. In order to mitigate residue levels and potential safety risks, it is advisable to implement a longer PHI or reduce the application rates of pesticides [48]. It is judged necessary to reassess the MRL or ‘guidelines for safe use’ for tebufenpyrad in angelica leaves. 

### 3.7. Confirmation of Pesticide Residues in Angelica Root

Angelica roots, known for their various phytochemicals with antioxidant and moisturizing effects, are primarily utilized as medicinal crops [49]. Despite foliage treatment, pesticides may persist in the roots through uptake and translocation from leaves, stem flow, or contact with fallen pesticides on the soil [50]. The precision and accuracy of the analytical methods in this study were ensured through recovery tests of penthiopyrad and tebufenpyrad in angelica roots (Table 6). 

Residue levels were tested in angelica roots harvested at each PHI in Fields 1–3 (Table 7). In each field, the average residue concentration of penthiopyrad in the angelica roots ranged from 0.33–1.57 mg/kg; for tebufenpyrad, it was within the range of 0.14–0.81 mg/kg. The sequence of penthiopyrad residue concentration at the PHI in angelica leaves was Field 3 > Field 1 > Field 2. The comparable pattern was obtained in the root residue concentration as well. The order of tebufenpyrad residue concentration at the PHI in angelica leaves was Field 1 > Field 3 > Field 2. The root residue concentration also exhibited the same pattern. The MRLs for penthiopyrad and tebufenpyrad in angelica roots have not been established. For crops that are consumed as roots (such as lance asiabell, radish, and bellflower), the MRLs for both pesticides are generally set at 0.05 mg/kg. Both pesticides exceeded the 0.05 mg/kg in all tested fields. In a South Korean distribution medicinal plant residue pesticide monitoring study, tebufenpyrad was detected at 0.16–0.4 mg/kg in cnidium [51]. In the domestic context, since there is no set MRL for tebufenpyrad in cnidium, the MRL should be calculated using the ‘guidelines for unestablished pesticide residues in medicinal crops’ [52]. According to the guidelines, the MRL for tebufenpyrad in cnidium is calculated to be 0.1 mg/kg. Tebufenpyrad in cnidium, a medicinal plant, exceeded the calculated MRL significantly. In Liu et al.’s study, residue levels of six pesticides (dichlofluanid, trifluralin, o,p′-DDT, trans-Permethrin, fenpropathrin, and parathon) in angelica roots met the registered MRL for each pesticide [53]. To ensure residues are managed at safe levels, it is important to establish and maintain standards (MRLs, ‘guidelines for safe use’, PHRLs, etc.). Therefore, it is determined necessary to set and assess standards for penthiopyrad and tebufenpyrad in angelica roots.

## 4. Conclusions

The PHRLs for penthiopyrad and tebufenpyrad, which are pyrazolecarboxamide pesticides, in angelica leaves were established. Samples were prepared following the simultaneous analytical methods for pesticide multi-residues (NO.2) in the Food Code by the MFDS in Korea. The analytical method was validated, and the LOQs for both pesticides were determined to be 0.01 mg/kg. The linearity of the calibration curve was satisfactory, with a correlation coefficient (*r*^2^) exceeding 0.999. The recovery and storage stability studies’ results were all within acceptable ranges (94.5–111.1%, RSD ≤ 9.1%), ensuring the accuracy and precision of the method. This analytical method was applied to the study of establishing PHRLs for penthiopyrad and tebufenpyrad in angelica leaves. After pesticide application, samples were harvested during certain periods and subjected to residue analysis. Both pesticides showed a dissipating trend in their residue concentrations over time following foliar application. Penthiopyrad 20 EC exhibited a half-life of 2.6–4.0 days, with an average of 3.1 days across the three fields. The proposed PHRL was ‘130.31 mg/kg at 10 days or 44.21 mg/kg at 5 days before harvest’. Tebufenpyrad 10 EC showed a half-life of 3.0–4.2 days, with an average of 3.7 days. The residue level (%) was at 293% on the PHI date; thus, the proposal for the PHRL has been deferred. It is determined essential to develop a new MRL or ‘guidelines for safe use’ for tebufenpyrad.

## Figures and Tables

**Table 1:** Retention times (t_R_) and established MRM conditions of penthiopyrad and tebufenpyrad.

Pesticide	t_R_ (min)	Monoisotopic Mass	Ionization Type	Precursor Ion > Product Ion (CE, V)	
				Quantifier	Qualifier
Penthiopyrad	3.69	359.1	[M+H]^+^	360.1 > 276.2 (−14)	360.1 > 177.1 (−32)
Tebufenpyrad	5.03	333.2	[M+H]^+^	334.2 > 117.1 (−37)	334.2 > 132.2 (−40)

**Table 2:** The method validation of penthiopyrad and tebufenpyrad for recovery and storage stability studies.

Pesticide	Study	Concentration (mg/kg)	Storage Period (Days)	Accuracy (%)	RSD ^(1)^ (*n* = 3) (%)
Penthiopyrad	Recovery	0.01	-	104.1	1.7
		0.1		94.5	2.0
		15		99.6	2.0
	Storage stability	1	25	97.0	9.1
Tebufenpyrad	Recovery	0.01	-	107.1	1.4
		0.1		108.1	1.1
		1		104.1	2.6
		20		111.4	2.0
	Storage stability	1	17	105.5	3.2

^(1)^ Relative standard deviation.

**Table 3:** The variation in tebufenpyrad residue levels in all three fields by date.

Pesticide	Day after Treatment (DAT)	Concentration (mg/kg)												
		Field 1				Field 2				Field 3				Field 1–3 Integration (Mean ± SD)
		Rep. 1	Rep. 2	Rep. 3	Mean ± SD ^(1)^	Rep. 1	Rep. 2	Rep. 3	Mean ± SD	Rep. 1	Rep. 2	Rep. 3	Mean ± SD	
Penthiopyrad	0	9.49	9.75	10.08	9.77 ± 0.30	8.82	8.92	10.55	9.43 ± 0.97	11.81	9.25	11.99	11.02 ± 1.53	10.07 ± 1.17
	1	6.76	7.37	9.17	7.77 ± 1.25	5.61	5.35	6.37	5.78 ± 0.53	11.43	10.85	9.31	10.53 ± 1.10	8.03 ± 2.25
	2	5.45	5.44	5.86	5.58 ± 0.24	5.53	4.76	5.63	5.31 ± 0.47	7.60	8.49	6.27	7.45 ± 1.11	6.11 ± 1.18
	3	5.12	4.78	5.29	5.06 ± 0.26	4.24	4.52	5.36	4.71 ± 0.58	6.95	5.59	7.02	6.52 ± 0.81	5.43 ± 0.98
	5	4.36	4.45	4.73	4.51 ± 0.20	2.41	1.83	1.91	2.05 ± 0.32	3.87	3.50	3.69	3.69 ± 0.19	3.42 ± 1.11
	7	1.87	1.86	2.21	1.98 ± 0.20	1.02	1.09	0.95	1.02 ± 0.07	2.24	2.46	2.69	2.46 ± 0.22	1.82 ± 0.65
	10	1.78	1.73	1.84	1.78 ± 0.06	0.78	0.74	0.69	0.74 ± 0.05	0.98	0.88	1.20	1.02 ± 0.17	1.18 ± 0.48
Tebufenpyrad	0	18.80	15.02	13.65	15.82 ± 2.66	14.73	16.76	15.35	15.61 ± 1.04	14.99	18.33	19.48	17.60 ± 2.33	16.35 ± 2.07
	1	13.32	12.20	11.78	12.43 ± 0.80	8.67	9.82	10.57	9.69 ± 0.96	15.35	15.34	17.61	16.10 ± 1.31	12.74 ± 2.93
	2	10.50	9.56	9.06	9.71 ± 0.73	5.96	6.90	6.83	6.56 ± 0.53	10.24	14.50	13.12	12.62 ± 2.17	9.63 ± 2.87
	3	9.34	8.56	7.19	8.36 ± 1.09	4.02	4.27	4.46	4.25 ± 0.22	10.43	8.24	9.23	9.30 ± 1.10	7.30 ± 2.45
	5	7.16	6.25	6.01	6.47 ± 0.61	2.23	2.31	1.64	2.06 ± 0.37	6.95	8.24	6.35	7.18 ± 0.97	5.24 ± 2.48
	7	4.68	3.70	4.69	4.36 ± 0.57	1.80	1.54	1.50	1.61 ± 0.16	3.42	2.44	2.56	2.81 ± 0.53	2.93 ± 1.26
	10	3.05	3.51	3.62	3.40 ± 0.30	1.30	1.64	1.48	1.47 ± 0.17	1.31	1.13	1.12	1.18 ± 0.11	2.02 ± 1.06
	14	1.20	1.37	1.44	1.34 ± 0.13	1.02	1.37	1.11	1.17 ± 0.18	1.20	1.04	1.06	1.10 ± 0.09	1.20 ± 0.16

^(1)^ Standard deviation.

**Table 4:** Pesticide dissipation regression analysis on angelica leaves.

Pesticide	Field	Dissipation Regression Equation ^(1)^	Regression Coefficient	95% CI ^(2)^ for Regression Coefficient ^(3)^	Dissipation Rate Constant
Penthiopyrad	1 (Sancheong)	y = 8.9487e^−0.1751x^ (*r*^2^ = 0.9353)	0.1751	0.1221–0.2280	0.1221
	2 (Jeonju)	y = 8.6264e^−0.2655x^ (*r*^2^ = 0.9658)	0.2655	0.2081–0.3230	0.2081
	3 (Jecheon)	y = 12.4510e^−0.2425x^ (*r*^2^ = 0.9911)	0.2425	0.2162–0.2689	0.2162
	1 to 3 (integrated)	y = 9.9338e^−0.2209x^ (*r*^2^ = 0.9905)	0.2209	0.1960–0.2457	0.1960
Tebufenpyrad	1 (Sancheong)	y = 14.5963e^−0.1646x^ (*r*^2^ = 0.9861)	0.1646	0.1451–0.1842	0.1451
	2 (Jeonju)	y = 9.1288e^−0.1810x^ (*r*^2^ = 0.8189)	0.1810	0.0960–0.2660	0.0960
	3 (Jecheon)	y = 18.2393e^−0.2274x^ (*r*^2^ = 0.9448)	0.2274	0.1725–0.2823	0.1725
	1 to 3 (integrated)	y = 14.0617e^−0.1891x^ (*r*^2^ = 0.9768)	0.1891	0.1600–0.2182	0.1600

^(1)^ Significant at 95% confidence level by the F-test. ^(2)^ Significant at 95% confidence level by the *T*-test. ^(3)^ Confidence Interval.

**Table 5:** Pesticide dissipation regression analysis on angelica leaves.

Pesticide	Pre-Harvest Residue Limit (mg/kg)												
	Days before Harvesting	10	9	8	7	6	5	4	3	2	1	0 (MRL)	Dissipation Rate Constant
Penthiopyrad	Field 1	50.87	45.02	39.84	35.26	31.21	27.62	24.45	21.64	19.15	16.95	15.00	0.1221
	Field 2	120.17	97.60	79.26	64.37	52.28	42.46	34.48	28.00	22.74	18.47	15.00	0.2081
	Field 3	130.31	104.98	84.57	68.13	54.88	44.21	35.62	28.69	23.11	18.62	15.00	0.2162
	1–3 (integrated)	106.44	87.50	71.93	59.13	48.61	39.96	32.85	27.00	22.20	18.25	15.00	0.1960
Tebufenpyrad	Field 1	4.27	3.69	3.19	2.76	2.39	2.07	1.79	1.55	1.34	1.16	1.00	0.1451
	Field 2	2.61	2.37	2.15	1.96	1.78	1.62	1.47	1.33	1.21	1.10	1.00	0.0960
	Field 3	5.61	4.72	3.97	3.34	2.81	2.37	1.99	1.68	1.41	1.19	1.00	0.1725
	1–3 (integrated)	4.95	4.22	3.60	3.06	2.61	2.23	1.90	1.62	1.38	1.17	1.00	0.1600

**Table 6:** The recovery rates of penthiopyrad and tebufenpyrad in angelica roots at 0.1 mg/kg.

Pesticide	Concentration (mg/kg)	Accuracy (%)			
		Rep.1	Rep.2	Rep.3	Mean ± RSD
Penthiopyrad	0.1	103.6	102.0	98.4	101.3 ± 2.6
Tebufenpyrad	0.1	89.5	103.2	108.9	100.5 ± 9.9

**Table 7:** Pesticides dissipation regression analysis on angelica leaves.

Pesticide	Accuracy (%)											
	Field 1 (Sancheong)				Field 2 (Jeonju)				Field 3 (Jecheon)			
	Rep.1	Rep.2	Rep.3	Mean ± RSD	Rep.1	Rep.2	Rep.3	Mean ± RSD	Rep.1	Rep.2	Rep.3	Mean ± RSD
Penthiopyrad	0.50	0.51	0.53	0.51 ± 0.01	0.30	0.31	0.37	0.33 ± 0.04	1.59	1.58	1.54	1.57 ± 0.03
Tebufenpyrad	0.80	0.89	0.72	0.81 ± 0.09	0.11	0.14	0.16	0.14 ± 0.02	0.30	0.31	0.41	0.34 ± 0.06

## Data Availability

The original contributions presented in the study are included in the article, further inquiries can be directed to the corresponding author.
